# A Specialist Herbivore Uses Chemical Camouflage to Overcome the Defenses of an Ant-Plant Mutualism

**DOI:** 10.1371/journal.pone.0102604

**Published:** 2014-07-21

**Authors:** Susan R. Whitehead, Ellen Reid, Joseph Sapp, Katja Poveda, Anne M. Royer, Amanda L. Posto, André Kessler

**Affiliations:** 1 Department of Ecology and Evolutionary Biology, University of Colorado at Boulder, Boulder, Colorado, United States of America; 2 Department of Biological Sciences, Louisiana State University, Baton Rouge, Louisiana, United States of America; 3 Department of Ecology and Evolutionary Biology, University of California at Santa Cruz, Santa Cruz, California, United States of America; 4 Department of Entomology, Cornell University, Ithaca, New York, United States of America; 5 Kellogg Biological Station and Department of Plant Biology, Michigan State University, Hickory Corners, Michigan, United States of America; 6 Department of Biology, Indiana University, Bloomington, Indiana, United States of America; 7 Department of Ecology and Evolutionary Biology, Cornell University, Ithaca, New York, United States of America; Universidade de São Paulo, Faculdade de Filosofia Ciências e Letras de Ribeirão Preto, Brazil

## Abstract

Many plants and ants engage in mutualisms where plants provide food and shelter to the ants in exchange for protection against herbivores and competitors. Although several species of herbivores thwart ant defenses and extract resources from the plants, the mechanisms that allow these herbivores to avoid attack are poorly understood. The specialist insect herbivore, *Piezogaster reclusus* (Hemiptera: Coreidae), feeds on Neotropical bull-horn acacias (*Vachellia collinsii*) despite the presence of *Pseudomyrmex spinicola* ants that nest in and aggressively defend the trees. We tested three hypotheses for how *P. reclusus* feeds on *V. collinsii* while avoiding ant attack: (1) chemical camouflage via cuticular surface compounds, (2) chemical deterrence via metathoracic defense glands, and (3) behavioral traits that reduce ant detection or attack. Our results showed that compounds from both *P. reclusus* cuticles and metathoracic glands reduce the number of ant attacks, but only cuticular compounds appear to be essential in allowing *P. reclusus* to feed on bull-horn acacia trees undisturbed. In addition, we found that ant attack rates to *P. reclusus* increased significantly when individuals were transferred between *P. spinicola* ant colonies. These results are consistent with the hypothesis that chemical mimicry of colony-specific ant or host plant odors plays a key role in allowing *P. reclusus* to circumvent ant defenses and gain access to important resources, including food and possibly enemy-free space. This interaction between ants, acacias, and their herbivores provides an excellent example of the ability of herbivores to adapt to ant defenses of plants and suggests that herbivores may play an important role in the evolution and maintenance of mutualisms.

## Introduction

Ant-plant mutualisms are a common and widespread feature of both temperate and tropical ecosystems [1], [2]. These interactions generally involve plants providing food and/or shelter to ants in exchange for protection from herbivores and competitors [3]–[5]. Ant-plant mutualisms involve diverse taxa, particularly in the tropics, including at least 100 plant genera and 40 species of ants that engage in interactions from generalized and facultative to highly specialized and obligate [6], [7]. These conspicuous examples of indirect plant defense are model systems for understanding plant defense theory, food web structure, species coexistence, plant/animal coevolution, and the evolutionary stability of mutualisms [8]. However, ant-plant mutualisms have most often been studied from the perspective of one or both of the interacting partners, and we still have a limited understanding of the community context of ant-plant interactions—how other organisms (herbivores, predators, etc.) interact with plants and their ant defenders [9]–[14].

Numerous studies have demonstrated that ants are a highly effective defense mechanism that protects plants against a broad range of potential herbivores [8], [15]. However, no plant defense is insurmountable [16], [17], and there are examples of herbivores that are able to feed on ant-plants despite the presence of aggressive defenders [9], [18]. These herbivores may gain important benefits from using ant-occupied plants as hosts, including enemy-free space, reduced competition, and access to food sources produced by the plant as a reward for the ant mutualists. Furthermore, the leaf tissues of ant-defended plants often have lower levels of chemical defenses relative to unoccupied plants [19]–[21], and thus are likely to be more palatable to herbivores. Gaining access to ant-defended plants requires specialized traits that reduce ant attack rates or increase tolerance to attack, and past evidence has suggested a variety of possible traits may serve this function. Examples include toughened exocuticles [18], [22], shelter-building behaviors [9], and behavioral avoidance maneuvers [23], [24]. An improved understanding of these and other potential adaptations to ant-defense systems can provide insights into the evolutionary dynamics and maintenance of ant-plant-herbivore interactions, specifically, and the exploitation of mutualistic interactions in complex communities, in general.

It is likely that herbivores of ant-plants will exhibit adaptations similar to those typical of other organisms that interact closely with social insects, either as parasites, predators, or mutualists. Thus, the subject of herbivory on ant-plants can be informed by insights from the large body of research on the ecology and evolution of both ant social parasites [reviewed in 25,26] and mutualists (e.g. ant-tended aphids) [27], [28]. Although ant societies use complex behavioral and chemical cues for nestmate recognition and colony defense [29]–[31], an estimated 10,000 species of social parasites from 11 invertebrate orders have evolved specialized mechanisms that allow them to exploit ant colony resources [29], [32]. These parasites use a variety of mechanisms to infiltrate ant colonies or access resources, including chemical mimicry, acoustic mimicry, behavioral adaptations, specialized morphologies, and defensive compounds that deter, agitate, or confuse workers [18], [26], [32], [33]. In particular, many organisms gain access to ant colonies through a variety of strategies that circumvent the ants' ability to identify intruders, which is accomplished primarily through chemical recognition of cuticular hydrocarbons [25], [31], [34]. These strategies can be broadly characterized as chemical camouflage, which we define here as any chemical strategy that prevents detection or recognition (after Stevens and Merilaita [35], but see Dettner and Leipert [34] for a discussion of alternative definitions). There are many examples of ant social parasites that achieve chemical camouflage by mimicking the cuticular hydrocarbon profiles of their ant hosts; however this mode of avoiding detection can carry the high cost of host specificity, so that the parasite individual or species is confined to living with just one species of ant or even just a single colony [36]–[38]. Chemical camouflage may also be achieved by mimicking other components of the environment in which the interaction takes place. For example, recent evidence has shown that ant-tended aphids can avoid ant aggression and predation by mimicking the chemical profile of the host plant on which the species interact [28]. A third form of camouflage used by some ant social parasites is “chemical insignificance”, when infiltrating organisms have minimal levels of cuticular compounds that are essentially undetected by ants [25], [39]. Herbivores of ant-plants could avoid detection and attack through any of these different chemical camouflage strategies, used either alone or in combination with other strategies, such as chemical defense (e.g. via repellent sprays) or behavioral adaptations (e.g. furtive or defensive movements).

**Figure 1:**
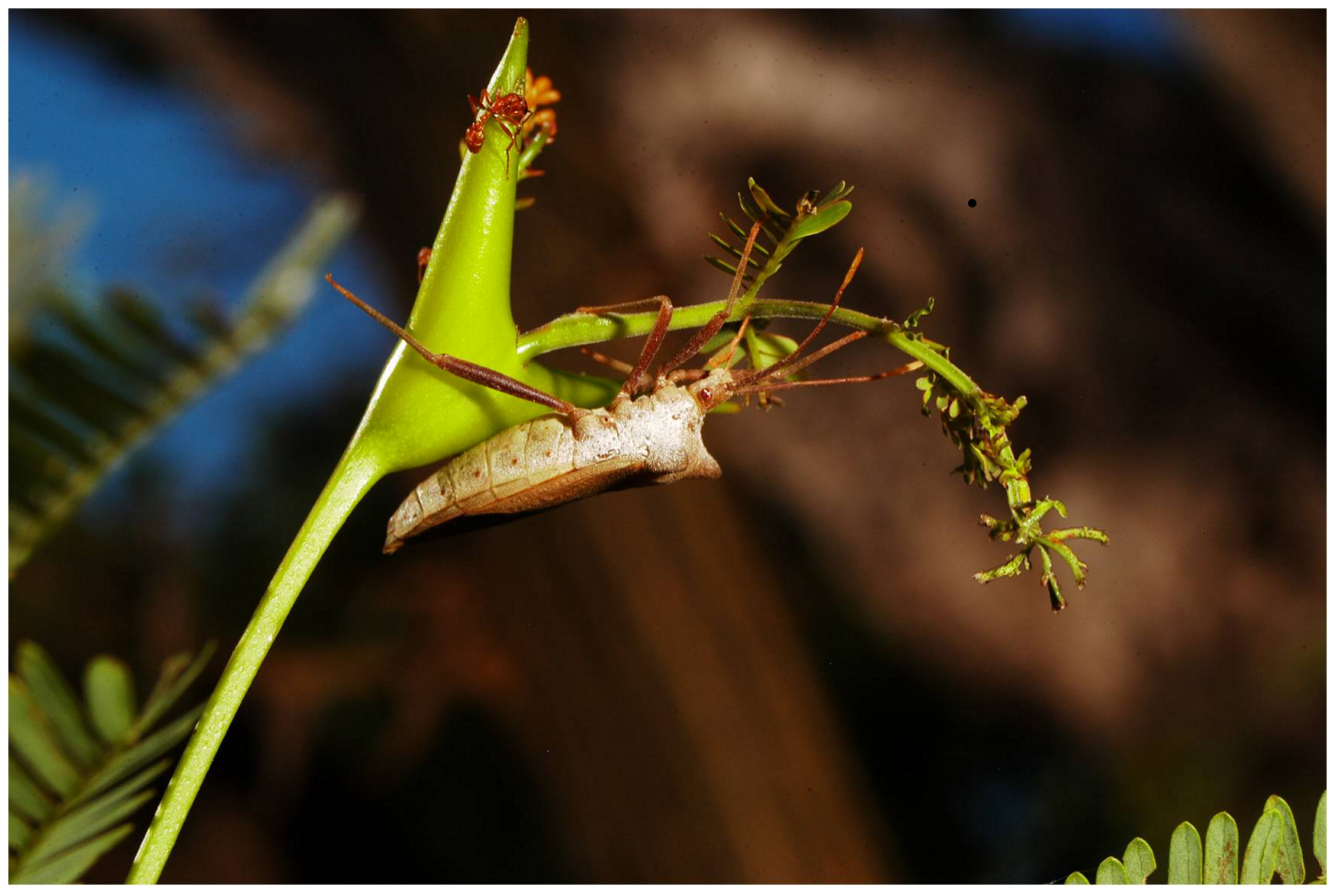
*Piezogaster reclusus* feeding on a *Vachellia collinsii* tree occupied by *Pseudomyrmex spinicola* ants. Photo by: André Kessler

Perhaps the most well-studied example of ant-plant mutualism is the interaction between *Pseudomyrmex spinicola* Emery 1890 (Hymenoptera: Formicidae) ants and Neotropical bull-horn acacias (*Vachellia collinsii* (Saff. 1950) Seigler & Ebinger 2005, formerly *Acacia collinsii*, hereafter referred to as acacias; [40]). Acacia trees provide food for the ants in the form of extrafloral nectaries and Beltian bodies (nutrient-rich leaf structures fed to ant larvae), as well as domatia in the form of hollow thorns that are used as nest chambers by the ants [8], [18], [41]. In return, the ants defend host trees from herbivores and pathogens and reduce competition by clearing away encroaching vegetation [8], [18], [41]. *Pseudomyrmex spinicola* workers are highly aggressive and swarm from domatia to bite and sting almost any foreign object that comes in contact with their host tree [41]. Despite this conspicuous defense, a sap-feeding coreid (*Piezogaster reclusus* Brailovsky & Barrera 2000, Hemiptera: Coreidae) feeds primarily on ant-occupied acacias, undisturbed by resident ants (Fig. 1, personal observation, [42]). Both adult and juvenile *P. reclusus* feed primarily on the sap of young leaves, causing the young leaves to wilt and resulting in the loss of leaf tissue, Beltian bodies, and extra-floral nectaries. Yet, despite the potential direct costs of this damage to both the plants and the resident ants, there is no apparent recruitment of ant defenders nor increase in aggressive ant behavior when *P. reclusus* feeds or walks on the trees.

In this study, we tested three non-mutually exclusive hypotheses to explain how *P. reclusus* is able to avoid ant attack on acacia trees. First, we hypothesized that *P. reclusus* avoids ant attack through chemical camouflage (e.g. chemical mimicry or insignificance), as do many other invertebrates that infiltrate ant societies [28], [29], [34]. Second, we hypothesized that *P. reclusus* avoids ant attack through chemical defenses that are repellent to the ants. Many species of hemipterans, including members of the family Coreidae, defensively spray highly concentrated volatile organic compounds from glands on the metathorax [43], [44], some of which bear resemblance to ant-alarm pheromones and may have evolved in response to ant predation pressure [44]. Thus, it is possible that the defensive sprays of *P. reclusus* may also provide an important line of defense against ant attack. Third, we hypothesized that *P. reclusus* avoids ant attack through behavioral traits. Preliminary observations in this and one previous short-term study [42] indicated that in cases where the coreids are attacked, they often respond by leg-lifting, where an attacked leg is lifted into the air and a quick flick dislodges the ant. Other behaviors may also be important in reducing detection by ants, such as walking softly to avoid movement of the branch that would cause ant alarm.

To test these hypotheses, we first conducted a series of field experiments designed to isolate the role of cuticular chemistry, glandular spray, and behavior in allowing *P. reclusus* to avoid ant attack. This involved examining the responses of *P. spinicola* ants to: 1) compounds extracted from *P. reclusus* cuticles or metathoracic glands, or 2) *P. reclusus* or other herbivores that do not feed on acacia trees, treated in various ways to isolate the effects of cuticle chemistry, glandular sprays, and behavior. Next, to further test the hypothesis of chemical camouflage, and to distinguish between different potential camouflage strategies (specifically mimicry versus chemical insignificance), we examined the host-specificity of *P. reclusus* on acacia trees occupied by different colonies of *P. spinicola*. Because effective chemical mimicry of ants or host plants would require *P. reclusus* to have cuticular hydrocarbon profiles that are colony-specific, we predicted that if the coreids avoided attack through chemical mimicry they would experience increased levels of ant aggression when moved to new host trees.

## Materials and Methods

### Study site

We conducted this study in Palo Verde National Park, Guanacaste Province, Costa Rica in Jan-Feb and March-April 2008, during the dry-season. The site is characterized as tropical dry forest (*sensu* Holdridge 1967) with an average annual rainfall of 1717 mm that occurs primarily during a distinct rainy season between mid-April and early November [45]. The vegetation is dominated by deciduous trees and shrubs, including *Allophyllus occidentalis* (Sapindaceae), *Astronium graveolens* (Anacardiaceae), and *Tabebuia ochracea* (Bignoniaceae) [45]. The bull-horn acacia, *Vachellia collinsii*, is a common understory tree that forms an obligate mutualism with one of three ant species, in order of most to least common at the study site: *Pseudomyrmex spinicola, P. flavicornis,* and *Crematogaster brevispinosa* [18].

### Ethics statement

All necessary permits for field research associated with this study were obtained from the Ministerio de Ambiente y Energía (MINAE, No. 01758, Resolución No. 048-2008-SINAC) in San Jose, Costa Rica with assistance from the Organization for Tropical Studies. The data collection and sampling did not involve endangered or protected species.

### General field bioassay methods

We conducted field experiments from 08:00 to 11:00 local time (GMT -6 hours) to standardize for level of ant activity, which tended to peak during the morning and decline in the afternoon (personal observation). During the study period the weather was sunny and no storms were observed. All *P. reclusus* individuals were collected opportunistically from acacia trees occupied by colonies of *P. spinicola* ants in forests bordering the park road near the Organization for Tropical Studies' Palo Verde Biological Station and held in vials for no more than 48 hours before completion of experiments. In our field experiments, we recorded ant responses to *P. reclusus* chemical cues on cotton swabs or insects receiving various treatments to isolate the effects of cuticular chemistry, glandular chemistry, and behavior. In all cases, the stimulus (cotton swab or insect) was placed on an ant-occupied tree near a young leaf that had extra-floral nectaries and Beltian bodies. With the exception of Experiment 3 (see below), insects or their extracted compounds were collected and later placed on haphazardly chosen trees at the study site, not necessarily those trees from which they were collected. We observed and recorded ant behavioral responses in continuous short trials that were 30 seconds to five minutes long, depending on ant activity. We recorded the number of attacks (ants that obviously stung or bit the stimulus) and the number of non-aggressive encounters (ants that encountered the stimulus, but either passed over or reversed direction without attacking). All field experiments (with the exception of Experiment 2b, see below) were conducted blind to the treatment—the researcher scoring ant responses was not aware of which treatment was being applied, and different treatments were placed in random order on separate branches within the tree. Replicate trials were conducted on different trees at least 25 meters apart to ensure they were occupied by different ant colonies.

### Ant responses to extracted chemical compounds

**Experiment 1a: “Cuticular compounds.”**

To test the role of *P. reclusus* cuticular chemistry in avoiding ant attack, we extracted cuticular surface compounds from live individuals of *P. reclusus* using lanolin paste and tested ant responses to these compounds. Lanolin is a natural wax derived from sheep wool and composed of esters, fatty acids, and alcohols that can be used to dissolve a variety of non-polar solutes [46]. We first prompted seventeen coreids to spray glandular compounds onto cotton swabs in order to deplete the contents of their glands. The glands were then sealed shut with clear nail polish and individuals were left for approximately one hour to allow the evaporation of volatile compounds from the glands. Each individual was then coated with lanolin paste on the exposed cuticle surfaces, including the dorsal and ventral side of the thorax and the ventral side of the abdomen. The lanolin-coated coreids were left overnight to allow the absorption of cuticular compounds by the lanolin paste. The following day lanolin paste from the coreids was transferred to cotton swabs. We then conducted paired trials on seventeen acacia trees, comparing ant responses to cotton swabs with either lanolin containing cuticular compounds or lanolin only (controls). Ant behavioral responses were recorded in 30-second trials as described above.

**Experiment 1b: “Glandular compounds.”**

To test the role of *P. reclusus* glandular spray in avoiding ant attack, we induced captured coreids to spray on cotton swabs in the field and placed these treated cotton swabs on ant-occupied trees. A separate individual of *P. reclusus* was used to treat a single cotton swab for each trial. We conducted seven paired trials, where each trial measured the responses of a single colony of *P. spinicola* ants to a glandular-treated and a control (dry) cotton swab. Ant behavioral responses were recorded in 30-second trials as described above.

### Ant responses to coreids (*P. reclusus*) and other herbivores

**Experiment 2a: “Cuticular washes.”**

To further test the role of *P. reclusus* cuticular chemistry in avoiding attack, we removed cuticular compounds by washing coreids in one of three solvents of varying polarity and placing them on to ant-occupied trees. Freshly-killed coreids were washed in one of three solutions: 100% distilled water (highly polar), 100% methanol (mid-polarity), and 100% hexane (highly non-polar). Insect cuticular hydrocarbons are highly non-polar [47] and should be completely removed by the hexane wash, partially removed by the methanol wash (depending on the specific compounds involved), and unaffected by the water wash. We washed individuals by soaking them for one hour in solvent and allowing them to dry for a minimum of one hour to allow evaporation of the solvent. Fifteen coreids were washed with each solvent treatment and then placed on acacia trees on their backs using a small amount of glue to ensure they remained on the branch for the duration of the trial. Each trial consisted of the placement of bugs from all three solvent treatments in random order on different branches and was conducted on a separate acacia individual occupied by a distinct colony. Ant behavioral responses were recorded in five-minute trials as described above in the general field bioassay methods.

**Experiment 2b: “Glandular spray and behavior.”**

To simultaneously test the roles of *P. reclusus* glandular spray and behavior in avoiding ant attack, we conducted a two-way factorial experiment with four treatment combinations. We first sealed the metathoracic glands of ten coreids with clear nail polish. Ten control individuals were painted with a similar quantity of clear nail polish on the prothoracic segment. We then placed individuals back onto acacia branches, randomly selecting half of the bugs from each nail polish treatment for a restraint treatment that would prevent typical coreid behaviors. Restraint was accomplished by gluing the bugs on their dorsal side to an acacia thorn. The remaining individuals were treated with a similar quantity of glue on their dorsal side, but were free to move about on the plants. As a positive control in this experiment, we also tested responses of the same ant colonies to other herbivores collected from the site that do not feed on acacia trees. These herbivores were collected opportunistically and included three individuals of an unidentified membracid (Hempitera: Membracidae), one individual of an unidentified weevil (Coleoptera: Curculionidae), and one individual of *Dysdercus sp.* (Hemiptera: Pyrrhocoridae). The control insects were restrained on the plant with a small quantity of glue in the same manner as the coreids. Each trial, consisting of the sequential placement of the control and all four treatments in random order on different branches, was conducted on a separate acacia individual occupied by a distinct colony. Ant behavioral responses were recorded in five-minute trials as described above.

**Experiment 2c: “Cuticular compound transfer.”**

To further test the role of *P. reclusus* cuticular compounds in avoiding ant attack, as well as the potential ecological relevance of cuticular chemistry in allowing colonization of acacia trees by other herbivores, we transferred lanolin paste containing *P. reclusus* cuticular compounds to other herbivores and placed them on ant-occupied trees. We collected 24 individuals of *Dysdercus sp.* (Hemiptera: Pyrrhocoridae), a common sap-feeding bug at our study site, often found in association with *Malvaviscus arboreus* (Malvaceae). Twelve of these insects were covered in lanolin containing *P. reclusus* cuticular compounds and twelve were covered with lanolin paste only as a control. Twelve behavioral trials were conducted on separate acacia trees, with each trial consisting of the placement of a randomly paired control and treated *Dysdercus* in random order on different branches. Ant behaviors were recorded in five minute trials as described above.

### Host specificity of coreids (*P. reclusus*)

**Experiment 3: “Host colony specificity.”**

To further test the chemical camouflage hypothesis, and specifically to distinguish between chemical mimicry (of ant or plant hosts) versus chemical insignificance, we conducted a translocation experiment that compared attack rates to coreids on the host tree from which they were collected versus new trees occupied by different ant colonies. We predicted that if chemical mimicry were the primary mechanism of camouflage, individuals of *P. reclusus* would experience increased aggression when moved among ant colonies. We collected ten adult *P. reclusus* individuals from ten different trees that were each occupied by a unique colony of ants, and held them in vials for one hour. Five live coreids were then translocated to new trees, each with a different colony of *P. spinicola,* and five were replaced on their trees of origin with the original colony of *P. spinicola*. Ant responses were recorded in 30-second trials as described above.

### Statistical analyses

For most experiments (1a-1b, 2a-2c), we examined the effects of treatment on ant behavior using generalized linear mixed models (GLMM) with the binomial distribution and the logit link function, fit by the Laplace approximation [48], [49]. These analyses were conducted using the ‘lme4’ package, Version 1.0–4 [50] in the R Environment for Statistical Computing, Version 2.15.3 [51]. Our response variables were the binomial counts of attack behaviors and non-attack (pass) behaviors. Treatment was always specified as a fixed effect and ant colony identity was always specified as a random effect. For hypothesis testing, we used likelihood ratio tests with Χ^2^ statistics that compared the full model to a null model that included the random effect only [52].

In the “cuticular washes” experiment (2a), we had three different treatments (hexane-washed, methanol-washed, and water-washed), and we followed the GLMM with pairwise comparisons of all treatments using a Tukey HSD *post-hoc* test. This was conducted using the ‘multcomp’ package in R, Version 1.3-0 [53]. For the “glandular spray and behavior” experiment (2b), there were only two out of 140 total encounters between ants and *P. reclusus* in which the coreids were attacked, thus we did not analyze the effects of gland and restraint treatment on ant attack rates statistically. However, we did statistically compare the attack rates to *P. reclusus* versus other control herbivores using a GLMM with herbivore identity (*P. reclusus* vs other) as a fixed effect and colony identity as a random effect. For this analysis, we included only the data for coreids that were restrained and had their glands closed.

For the “host colony specificity” experiment (3), we used a different ant colony for each trial, so we compared ant responses to translocated versus replaced treatments using a generalized linear model (GLM) in R with no random effects. We used the binomial distribution with the logit link function, and the proportion of ant attacks was specified as a binomial response variable as above.

## Results

### Ant responses to extracted chemical compounds

**Figure 2:**
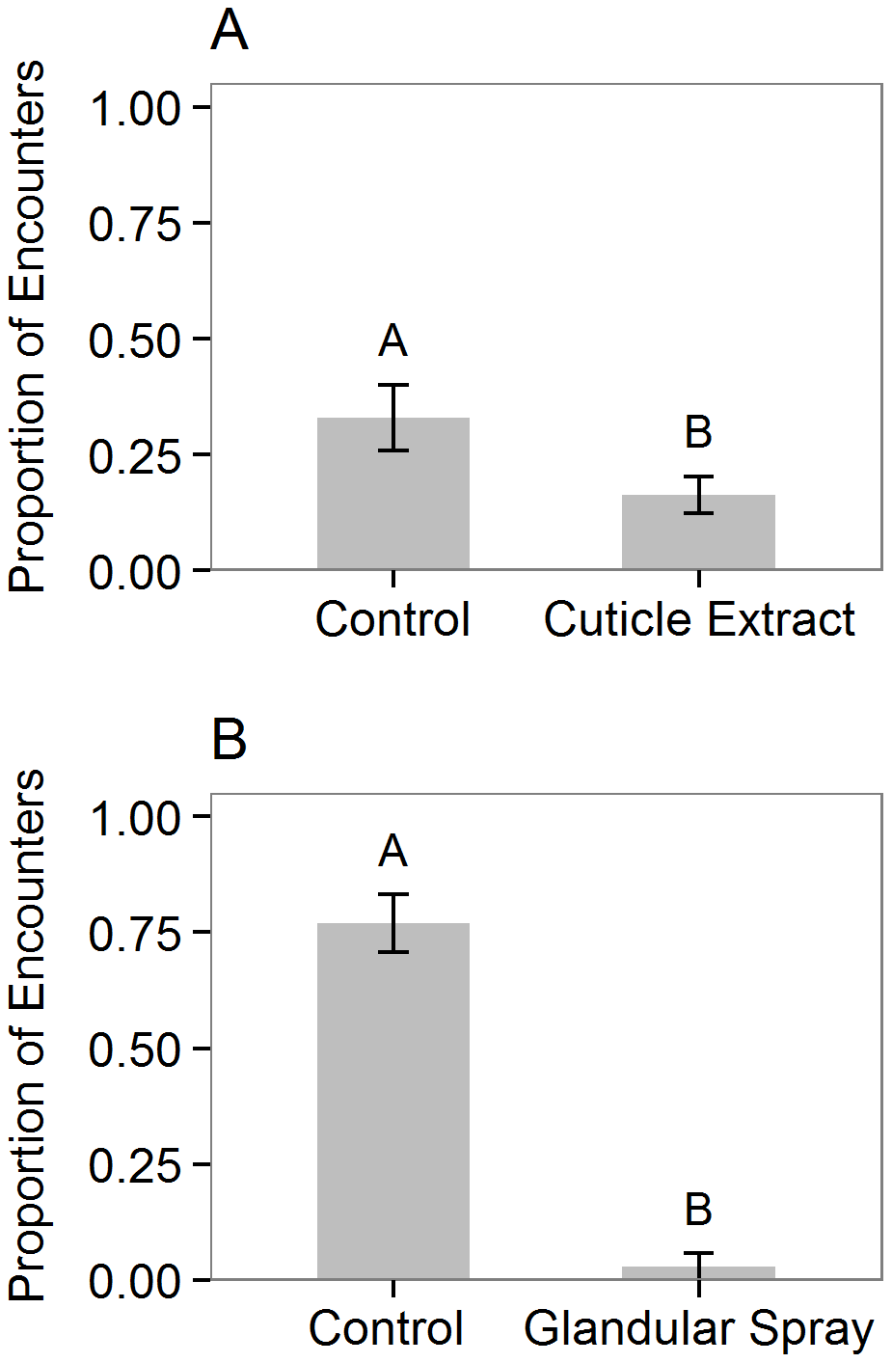
Response of *Pseudomyrmex spinicola* ants to isolated compounds from *Piezogaster reclusus*. Bars show the mean proportion (± SE) of encounters in which *Pseudomyrmex spinicola* ants attacked cotton swabs that were treated with *Piezogaster reclusus* (A) cuticular compounds or (B) glandular compounds in comparison to controls. Letters indicate significant differences from binomial GLMMs that included ant colony as a random effect.

In the “cuticular compounds” experiment (Exp. 1a), cotton swabs treated with extracted cuticular compounds were attacked in 16% of encounters, significantly less often than lanolin-only controls, which were attacked in 33% of encounters (GLMM; Χ^2^ = 12.73, df = 1, p = 0.00036; Fig. 2A). In the “glandular compounds” experiment (Exp. 1b), cotton swabs treated with glandular compounds were attacked in 3% of encounters, significantly less often than controls, which were attacked in 77% of encounters (GLMM; Χ^2^ = 51.51, df = 1, p<0.0001; Fig. 2B).

### Ant responses to coreids and other herbivores

**Figure 3:**
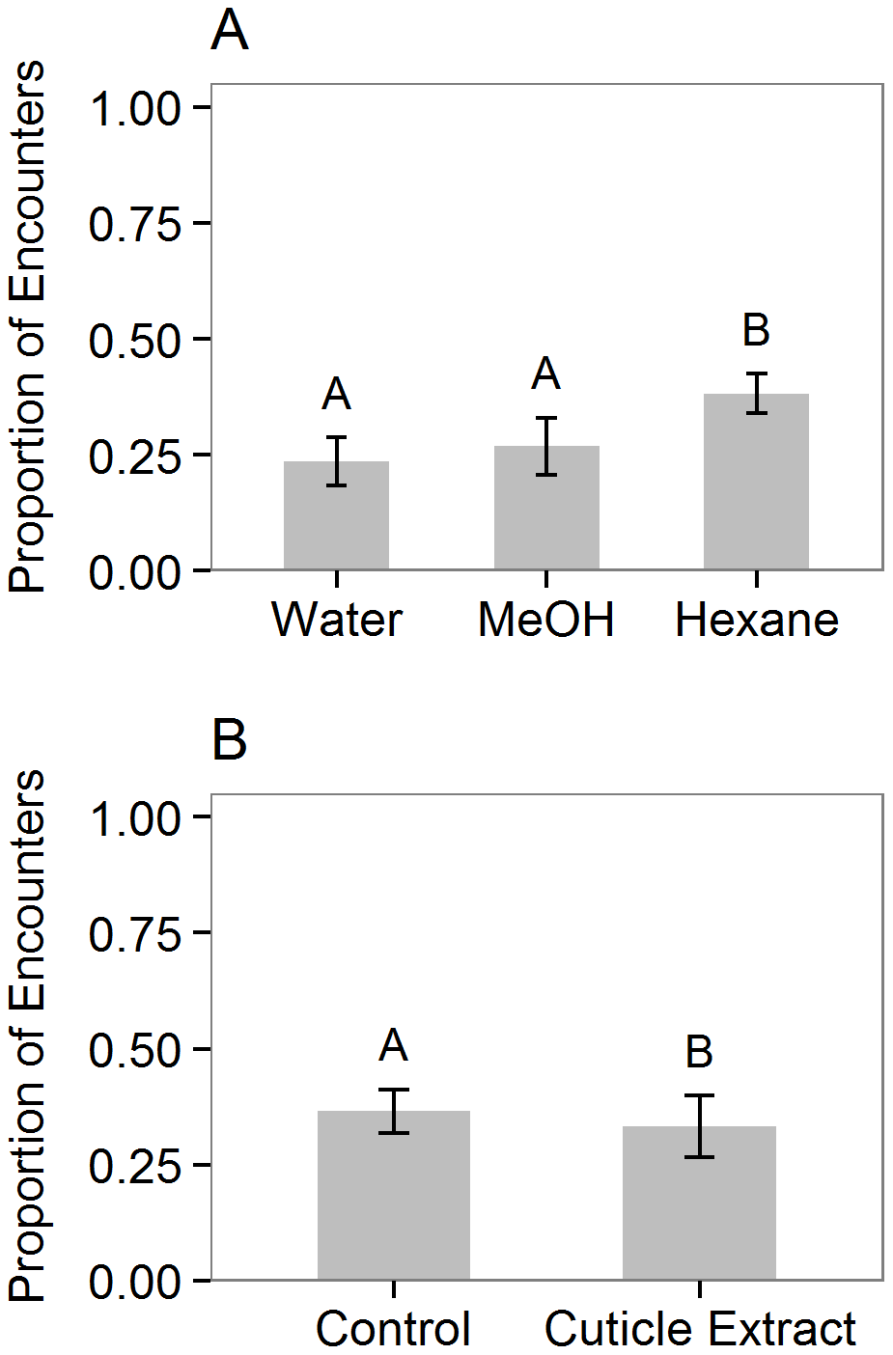
Response of *Pseudomyrmex spinicola* ants to *Piezogaster reclusus* and other herbivores. Bars show the mean proportion (± SE) of encounters in which *Pseudomyrmex spinicola* ants attacked: (A) dead individuals of *Piezogaster reclusus* that were washed in water, methanol, or hexane to remove cuticular compounds, or (B) individuals of *Dysdercus sp.* that were treated with lanolin-extracted *Piezogaster reclusus* cuticular compounds or lanolin only as a control. Letters indicate significant differences from binomial GLMMs that included ant colony as a random effect.

In the “cuticular washes” experiment (Exp. 2a), water-washed coreids were attacked in 24% of encounters, MeOH-washed coreids were attacked in 27% of encounters, and hexane-washed coreids were attacked in 38% of encounters. There was an overall effect of wash treatment on the proportion of ant attacks (GLMM; Χ^2^ = 16.20, df = 2, p = 0.00030). *Post-hoc* comparisons among treatments showed that hexane-washed coreids were attacked significantly more often than water-washed (p = 0.00053) or methanol-washed (p = 0.0072) coreids (Fig. 3A). There was no significant difference between methanol-washed and water-washed coreids.

In the “glandular spray and behavior” experiment (Exp. 2b), we conducted five replicate trials with 140 total ant encounters, and observed only two occurrences of an ant attacking *P. reclusus*, both on the same unrestrained individual with open glands. Other restrained herbivores were attacked, on average, in 39% of encounters, which was significantly more often than coreids that were restrained with their glands sealed (GLMM; Χ^2^ = 23.17, df = 1, p<0.0001).

In the “cuticular compound transfer” experiment (Exp. 2c), individuals of *Dysdercus sp.* treated with lanolin containing coreid cuticular compounds were attacked in 33% of encounters, significantly less often than those treated with lanolin only, which were attacked in 37% of encounters (GLMM; Χ^2^ = 4.60, df = 1, p = 0.032; Fig. 3B).

### Host specificity of coreids (*P. reclusus*)

**Figure 4:**
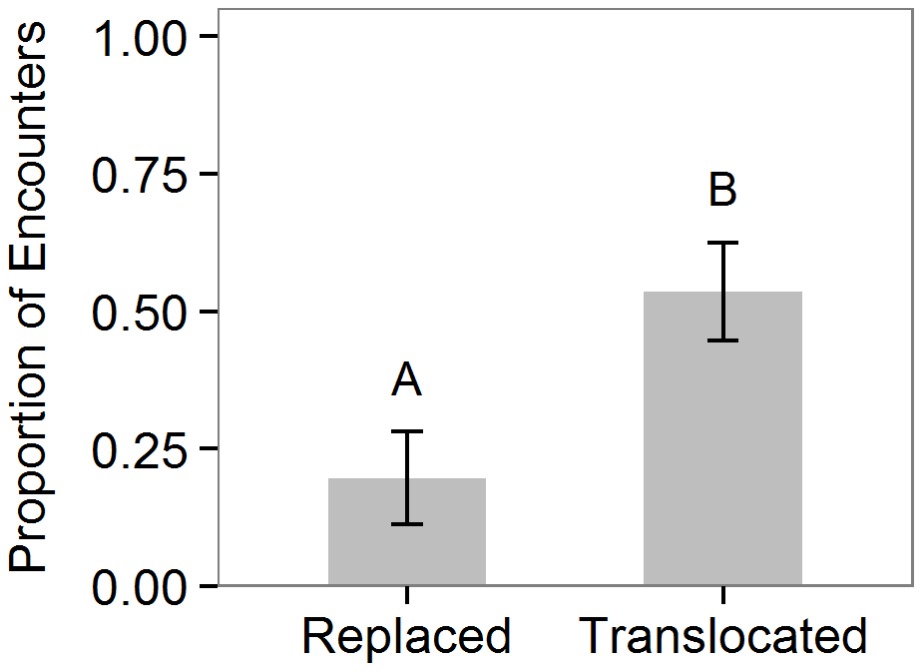
Response of *Pseudomyrmex spinicola* ants to replaced or translocated individuals of *Piezogaster reclusus.* Bars show the mean proportion (± SE) of encounters in which *Piezogaster reclusus* was attacked when replaced on the host tree from which it was collected or translocated to a new tree with a different ant colony. Letters indicate significant differences from a binomial GLM.

In the “host colony specificity” experiment (Exp.3), coreids translocated to new trees occupied by different *P. spinicola* ant colonies were attacked in 54% of encounters, significantly more often than coreids replaced on their tree of origin, which were attacked in 20% of encounters (GLM; *z* = 2.39, df = 8, p = 0.017; Fig. 4).

## Discussion

In the classic example of ant-plant mutualism between Neotropical bull-horn acacias and *Pseudomymex* ants, a number of herbivores have been described that feed on acacias undisturbed by resident ants [9], [18], [23], [54]. However, the mechanisms by which these organisms are able to circumvent the highly specialized defense system of the ants are often poorly understood. Our results show that non-polar compounds present on the cuticles of a coreid bug, *P. reclusus*, provide protection against attack from *P. spinicola* ants. Other mechanisms of avoiding attack, such as specialized behaviors or deterrent compounds sprayed from the coreid metathoracic gland, may also play a role in reducing attack, but the coreids' ability to feed among ants seems to persist even when these traits are experimentally removed. Furthermore, our experiments show that *P. reclusus* bugs experience increased levels of attack when transferred among ant colonies, suggesting that the protection provided by coreid cuticular chemistry is colony-specific. Although many questions remain about the specific chemical cues involved and their mode of action, our results provide the most support for the hypothesis that chemical camouflage, in general, and chemical mimicry, in particular, of either ant or plant cuticular hydrocarbons is a key mechanism that allows *P. reclusus* to feed on acacia trees while avoiding attack.

Results from our field experiments with extracted cuticular compounds, coreids receiving various wash treatments, and other insects with coreid cuticular compounds transferred to their cuticles (Fig 2A, Fig. 3A, Fig. 3B, respectively) show that the compounds present on coreid cuticles provide protection against ant attack and are consistent with the hypothesis that these compounds mimic either compounds present on ant cuticles or on the plant surface. Lanolin extracts of non-polar cuticular compounds from live coreids reduced ant attack rates when presented on cotton swabs (Fig. 2A) and also provided some protection for other herbivorous insects (*Dysdercus sp.*) when transferred to their cuticle surfaces (Fig. 3B). Furthermore, coreids whose cuticular chemistry had been altered/removed via hexane washes were attacked significantly more than those washed in MeOH or water (Fig. 3A). Although we expected that MeOH washes would also remove some cuticular hydrocarbons and increase attack rates relative to water-washed insects, there was only a slight, non-significant trend towards increased attack on MeOH-washed relative to water-washed insects. It is likely that the methanol was less efficient than hexane at removing the relevant hydrocarbons from the cuticle surface, suggesting highly non-polar cuticular hydrocarbons as the putative mimicry signal. Thereby *P. reclusus* cuticular chemistry could mimic either ant or plant cuticular hydrocarbon composition or both. To our knowledge the cuticular hydrocarbons present on *P. spinicola* have not been identified, but the profiles of closely related species of *Pseudomyrmex* are dominated by highly non-polar mono- and dimethyl-alkanes varying in chain length from C27 to C37 [55]. Even less is known about acacia surface chemistry, which equally likely could provide cues either for the *P. reclusus* bugs to mimic the host plant and therefore be undetected by ants, or for ants and bugs to express similar cuticular chemistry as a result of similar dietary intake [56], [57].

Our experiments also partially supported our second hypothesis, that defensive compounds produced in the coreid methathoracic glands reduce the level of ant attack. Glandular compounds on cotton swabs resulted in a nearly 30-fold decrease in the proportion of encounters that were attacks (Fig. 1B), providing strong evidence that these compounds are deterrent to ants. However, our experiments with coreids that had their glands experimentally closed indicated that coreids are still rarely attacked by ants even without their ability to use glandular sprays as a defense. The fact that ants were active in the experiment (with 140 total encounters with coreids) and were aggressive towards other herbivores suggests that glandular defensive spray is non-essential in avoiding attack by ants on the host tree. Furthermore, our results showing that sealing glands has no effect on ant attack rates were similar to results found in a preliminary study [42]. However, these results do not preclude the possibility that glandular sprays may be used as an important line of defense when coreids are attacked. Interestingly, in our experiment with glandular compounds on cotton swabs, we observed a strong tendency for ants that encountered the treated swabs to avoid the stimulus, usually backing away and changing direction, rather than passing by and continuing in the same direction as they often did when the stimulus involved a coreid or extracted cuticular compounds. This behavior suggests that ants were in fact detecting the glandular compounds, but were repelled by the odor rather than initiating typical defense behaviors. Other species in the family Coreidae are known to use volatile glandular compounds in defense [44], and these chemicals may have a generalized deterrent function against ants as well as other predators or natural enemies.

Our third hypothesis, that behaviors such as leg-lifting or walking softly can also play a role in protecting coreids against ant attack, was not supported in this study. Our treatment of restraining coreids by gluing them to the plant clearly prevented normal behaviors, but led to no detectable increase in ant attack. However, when coreids were free to move around on the plant, we did observe the leg-lifting behavior as noted in an earlier study [42]. Our results do not preclude the possible importance of these and other behaviors on occasions when ants do attack coreids, but suggest that, like the glandular spray, behavior is not the primary mechanism through which coreids are able to persist on acacia trees.

Our final set of experiments was designed to further test the hypothesis of chemical mimicry by determining the host specificity of coreids to different colonies of *P. spinicola*. Because cuticular hydrocarbons are used by ants in recognition at the nestmate level, we predicted that if coreids do use chemical mimicry to avoid attack, individuals would be specialized with respect to ant colony. We did find that coreids were attacked more often when translocated to new colonies of *P. spinicola* (Fig. 4). Other explanations for how cuticular hydrocarbon chemistry may be involved in reducing attack, such as the “chemical insignificance” hypothesis [25], [39] or the possibility that coreid cuticular compounds are somehow deterrent or confusing to the ants, would predict no change in ant aggression when coreid bugs are moved from home ant colonies to foreign ant colonies. Thus, these results provide important additional support for the hypothesis of chemical mimicry and also have implications for understanding the constraints imposed by this strategy in terms of host plant use and dispersal by *P. reclusus*.

It is unclear whether coreids synthesize cuticular hydrocarbons or acquire them from the host ant colony or host plant. Because the entire life cycle of *P. reclusus* is associated with acacia trees, including egg and nymph stages (personal observation), one possibility is that host colony odors are acquired by the coreids early in development and single individuals spend the majority of their lives on a single tree. Reproductive individuals may later disperse to trees occupied by different colonies, and although our results suggest host-switching may be initially costly in terms of increased attack rates, coreids may acquire host colony or host plant odors over time and eventually feed on the new tree undisturbed. Even if adult coreids do not acquire new host colony odors, they may be able to avoid ant attack through a combination of chemical deterrence and behavior for long enough to mate and lay eggs on a new tree. Future work examining the composition and changes in cuticular hydrocarbon profiles during *P. reclusus* development and how these changes relate to the dispersal ability of the coreids among ant colonies and host trees will help to identify the potential costs involved in chemical mimicry and its effects on *P. reclusus* demography.

Our study provides the first description of the mechanisms by which a coreid (*P. reclusus*) is able to overcome the ant defenses of the Neotropical bull-horn acacias (*Vachellia collinsii*), and our results are consistent with the hypothesis that coreids use chemical mimicry to avoid aggression from the ants. Future work in this system should seek to: 1) characterize the cuticular hydrocarbon profiles of ants, coreids, and their common host plants, 2) determine whether coreid cuticular hydrocarbons are synthesized *de novo* or acquired from ant colonies or host plants, and 3) further examine the dispersal of coreids among host trees and the variation in ant-acacia-coreid interactions in space and time. The ant-acacia interaction is a textbook example of mutualism that is considered to be highly specialized and tightly coevolved [8], [41]. However, herbivores are able to overcome the ability of ants to effectively defend their host trees, and exploitation of the chemical recognition system of the ants is one important way through which this can occur. An improved understanding of the mechanisms and outcomes of these interactions can provide new insights into the effectiveness of biotic defense against herbivory and the evolution and maintenance of mutualistic interactions.
